# Small ruminant macrophage polarization may play a pivotal role on lentiviral infection

**DOI:** 10.1186/1297-9716-44-83

**Published:** 2013-09-26

**Authors:** Helena Crespo, Luigi Bertolotti, Magda Juganaru, Idoia Glaria, Damián de Andrés, Beatriz Amorena, Sergio Rosati, Ramsés Reina

**Affiliations:** 1 Instituto de Agrobiotecnología, CSIC-Universidad Pública de Navarra, Mutilva Baja, Navarra, Spain; 2 Dipartimento di Produzioni Animali, Epidemiologia ed Ecologia, Università di Torino, Torino, Italy

## Abstract

Small ruminant lentiviruses (SRLV) infect the monocyte/macrophage lineage inducing a long-lasting infection affecting body condition, production and welfare of sheep and goats all over the world. Macrophages play a pivotal role on the host’s innate and adaptative immune responses against parasites by becoming differentially activated. Macrophage heterogeneity can tentatively be classified into classically differentiated macrophages (M1) through stimulation with IFN-γ displaying an inflammatory profile, or can be alternatively differentiated by stimulation with IL-4/IL-13 into M2 macrophages with homeostatic functions. Since infection by SRLV can modulate macrophage functions we explored here whether ovine and caprine macrophages can be segregated into M1 and M2 populations and whether this differential polarization represents differential susceptibility to SRLV infection. We found that like in human and mouse systems, ovine and caprine macrophages can be differentiated with particular stimuli into M1/M2 subpopulations displaying specific markers. In addition, small ruminant macrophages are plastic since M1 differentiated macrophages can express M2 markers when the stimulus changes from IFN-γ to IL-4. SRLV replication was restricted in M1 macrophages and increased in M2 differentiated macrophages respectively according to viral production. Identification of the infection pathways in macrophage populations may provide new targets for eliciting appropriate immune responses against SRLV infection.

## Introduction

Small Ruminant Lentivirus (SRLV) infection is spreading all over the world, with new descriptions in countries such as Poland [1], Sultanate of Oman, Canada [2], Slovenia [3], Russia [4] and new genotypes such as A12, A14 and A15 [3], E and B3 [5-7]. Today, highly efficient prophylactic/therapeutic measures against SRLV do not exist, and control is frequently based on early diagnosis and culling of seropositive ewes and their progeny. SRLV infection occurs early after parturition by the lactogenic route or direct contact with infected animals. The main target cells for the virus in vivo are the monocyte/macrophage lineage [8] whereas CD4 T cells and dendritic cells also play important roles [9,10]. Upon infection, initial viral replication triggers T cell and antibody responses that control virus burst and allow serological diagnosis. Following this stage, viral infection is partially controlled but provirus is already integrated at low levels into the cellular genome mainly of monocytes, myeloid cells or tissue macrophages [11]. The provirus remains latently integrated until viral proteins are produced and new virions are released. This results in a continuous process of low viral replication together with expression of pro-inflammatory cytokines and chemokines that often lead to a strong inflammatory process, tissue damage and disease development [11]. The permissiveness of macrophages to SRLV in vitro is modulated by cytokines. Specifically, IFN-γ restricts SRLV replication by delaying macrophage maturation [12-14], whereas increased IL-8, GM-CSF, IL-16, IL-1beta, IL-4 and IL-10 have been associated in vivo to seropositivity [15-18] and SRLV replication in vitro.

Cells of the monocyte-macrophage lineage are heterogeneous reflecting the plasticity and versatility of the response to environmental stimuli required for effective immune responses. Different patterns of macrophage maturation have been identified in humans and mice according to differentiation mechanisms and identification of markers for such subpopulations [19]. Macrophage classical (M1 phenotype) and alternative activations (M2 phenotype) depend on the presence of molecules secreted by T-helper CD4 or NK cells [20]. In particular, M1 phenotype is induced by Th1-signature cytokines such as IFN-γ and TNF-α as well as LPS, resulting in enhanced microbicidal ability in addition to increased proinflammatory responses and cellular immunity [21]. In HIV-1 infection, the M1 profile is characterized by down-regulation of CD4 receptors, increased CCR5-binding chemokines and significantly decreased viral production, likely at pre-integration steps [22]. On the contrary, the M2 phenotype is displayed after induction with Th2-hallmark cytokines such as IL-4 and IL-13, which play a major role in responses against parasites, allergy, wound healing, tissue remodeling or in some cases to regulate the immune response [23].

In contrast with humans and mice, data on macrophage polarization and its effect on lentiviral infection are lacking in other animal species. This study aimed to investigate whether sheep and goats, both targets to SRLV infections, also display macrophage subpopulations. For this, we explored putative stimuli that would elicit such subpopulations and determined cell markers to identify them. With the information obtained, we investigated if the putative subpopulations had different permissiveness and effectiveness of infection by different strains of SRLV, according to performance in an entry assay and retrotranscriptase activity, respectively.

## Materials and methods

### Cells and viruses

Fibroblastic-like cells from skin biopsies were obtained from SRLV-free animals. Cells were grown in DMEM medium supplemented with 10% foetal bovine serum, 2% L-glutamine, 1% antibiotics/antimycotics mix and 1:1000 gentamicin (Sigma-Aldrich, Steinheim, Germany).

Blood monocyte-derived macrophages (MDM) were obtained by isolation of peripheral blood mononuclear cells (PBMC) from SRLV-free sheep and goats in compliance with the relevant National legislation on experimental animals and animal welfare, upon authorization by the competent authority (Italian Ministry of Health-Directorate General Animal Health-Office VI, permit no.07/2009B), by Ficoll (1.077) gradient centrifugation. MDM were grown in RPMI complete medium, containing RPMI 1640 supplemented with 10% foetal goat serum, 10 mM sodium pyruvate, 1% non-essential amino acids, 1% vitamins, 1% antibiotics/antimycotics mix and 1:1000 gentamicin, 1% L-glutamine and 50 μM 2-mercaptoetanol (Sigma-Aldrich).

HEK293-T cells were cultured in DMEM (GIBCO, Invitrogen, Paisley, UK) supplemented with 10% foetal bovine serum, 1% antibiotics/antimycotics and 1:1000 gentamicin.

CHO cells and CHO-MR cells expressing mouse mannose receptor (kindly provided by Dr Luisa Martínez-Pomares; [24]) were cultured in F12 nutrient mixture with 10% foetal bovine serum, 1% L-glutamine, 1% antibiotics/antimycotics and 1:1000 gentamicin.

Six different SRLV strains were used, one from the genotype A VMV-like Ev1 strain [25], three of them belonging to the genotype B CAEV-Co [26] and CAEV-To1/89 (B1, [27]), and 496 (B2, [28]) and two belonging to the genotype E, Roccaverano (E1, [5] and Seui (E2, [6]).

### Cytokine expression

Different small ruminant cytokines were used as well as LPS (Sigma) as stimulators for macrophage polarization. Plasmid pN3-IFN-γ containing the ovine IFN-γ gene was kindly provided by Dr Marie Suzan-Monti (Faculté de Médecine, Marseille, France).

Primer design for caprine IL-4, IL-13 and IL-10 cloning (Table 1) was based on sequences available at Genbank (accession numbers U34273.1, NM_001082594 and U11421, respectively). Primers contained specific restriction sites (*XhoI* and *Acc651*) for posterior subcloning into the pN3 eukaryotic expression vector. pN3 is derived from pN3-EGFP (Clontech Laboratories, Saint-Germain-en-Laye, France) from which the EGFP gene was removed [29]. All the forward primers contained the Kozak consensus sequence ACC. ATG.G.

**Table 1:** Primers used for amplification and cloning of the cytokines and the Caev-Cork env gene used in entry assays.

**Molecule**	**Primer**		**Probe**	**Amplicon length**
	**Forward**	**Reverse**		
IL-4	AATTCTCGAG**ACCATGG**GTCTCACCTCCCAGC	AATTTGGTACCTCAACACTTTGAGTATTTCTCC		472 nt
IL-13	AATTCTCGAG**ACCATGG**CGCTCTTCTTGAC	ATTTGGTACCTCAGTTGTAACTTCCATTGCG		556 nt
IL-10	AATTCTCGAG**ACCATG**CCCAGCAGCTCAG	ATTTGGTACCTTACATCTTCGTTGTCATGTA		419 nt
CAEV-Cork *env*	ATATAGATCTCCACCATG	ATTTGCGGCCGCTATTAGTCCTCTTTAG		2834 nt
TNF-α qPCR	GGTGCCTCAGCCTCTTCTC	GAACCAGAGGCCTGTTGAAG	6-FAM-TGGTTGCAGGAGCCACCACG-TAMRA	134 nt
CD80 qPCR	CTGTGATTACAACACGACCACTGA	ATGGTGCGGTTCTCGTATTCA	6-FAM-AACTGGCAAGCCTTCGGATCTACTGGC-TAMRA	128 nt [30]
A3Z1 qPCR	TCCGTTCTTGGAATCTGGAC	GTATAGATGCGGGAGGCAAA		151 nt
MR qPCR	TGGCAAATCCAGTTGTTAAGATGTT	AGAATGTTGAATACTGTGGCGAGTT		91 nt [31]
DC-SIGN qPCR	GGTTCCGGAGTCTGACTGAAGTT	GGTCAGGCGCTGTAGGATCTC		73 nt
IL-10 qPCR	CGGCGCTGTCATCGTTTT	TCTTGGAGCATATTGAAGACTCTCTTC	6-FAM-CCTGCTCCACCGCCTTGCTCTTG-TAMRA	82 nt

Sequences are listed 5′to 3′; restriction enzyme recognition sites are underlined; Kozak sequences are in bold. A3Z1, MR and DC-SIGN were amplified using SybrGreen Master Mix (Takara).

Primers and probes (when corresponding) for relative expression quantification using real time PCR (qPCR) are also indicated.

Polymerase chain reactions (PCR) were performed in a final volume of 50 μL with 3 μL of the cDNA samples. The reaction mixture contained 1× PCR buffer, 2 μM dNTP, 3 nM of each primer and 1.25 units of Hot Start Polymerase (Qiagen, Hilden, Germany). The PCR started with a 95 °C step (15 min), followed by a denaturation step at 94 °C for 30 s, 1 min of annealing at 55 °C and a 1 min extension at 72 °C. A final extension of 72 °C for 10 min was also carried out.

PCR products were submitted to electrophoresis in a 1.5% agarose gel, and the selected amplicons were purified by Qiagen PCR clean-up kit, digested with *XhoI/Acc561* restriction enzymes and cloned into the previously digested expression vector pN3. Chemically TOP10 competent cells (Invitrogen, Paisley, UK) were transformed and grown overnight in LB agar plates supplemented with kanamycin (50 ng/mL). Colonies were screened by PCR with specific primers for the vector sequences, run in 1.5% agarose gel and positive clones were selected and sequenced (Secugen, Madrid, Spain).

Plasmids containing the correct IL-4, IL-13 or IL-10 sequence were employed for transfection of cultured HEK 293-T cells using the Amaxa Nucleofector II device and following the manufacturer’s protocol from Cell Line Nucleofector Kit V (LONZA, Köln, Germany). After 72 h, cell supernatants were collected, clarified by centrifugation and frozen at −80 °C. HEK 293-T cells were also transfected with the empty plasmid pN3 and the supernatant was used as a non-stimulated control.

### Quantification of cytokines for use as stimulators in macrophage polarization

IFN-γ and IL-4 cytokine concentrations and biological activities in HEK 293-T culture supernatants was measured with the Ovine IFN-γ ELISA kit (Mabtech, Nacka Strand, Sweden) and the Bovine IL-4 screening set (Thermo Scientific, Rockford, USA), respectively, according to the manufacturers’ protocols. The concentration obtained was similar in both cases, ranging from 600 to 2600 ng and was adjusted to 50 ng/mL for use in MDM stimulations (see below).

In the absence of commercially available quantification kits for small ruminant IL-10 and IL-13, the concentration range of these cytokines in the HEK 293-T culture system was assumed to be the same as for IFN-γ and IL-4. Nevertheless, biological activity was first verified by addition of 5, 10 and 20-fold dilutions of the HEK 293-T culture supernatants to MDM, followed by a 4 h incubation, cell harvesting, RNA extraction and determination of marker expression by real time RT-PCR (see below).

### Macrophage polarization

LPS and cytokines (IFN-γ, IL-4, IL-13 and IL-10) were added to macrophages on day 3 of culture, at a final concentration of 50 ng/mL. Supernatants from HEK 293-T cultures transfected with empty pN3 plasmid were used as the negative control. Fresh medium supplemented with cytokines was partially replaced after 3–4 days of culture. On day 6, stimulated MDM were either collected for RNA extraction and marker molecule expression analysis (at least six independent wells for each treatment), or infected for RT activity measurements or used in entry assay experiments, as indicated below.

### Marker expression analysis by real time RT-PCR

RNA was extracted from cytokine treated and pN3-treated MDM and real time RT-PCR were carried out using specific primers and probes according to the differentiation pathway, TNF-α, CD80 and APOBEC3Z1 (A3Z1) for M1 phenotype and Mannose Receptor (MR), DC-SIGN and IL-10 for M2 phenotype (Table 1). TNF-α, CD80 and IL-10 real time PCR procedures were performed using specific probes and conditions described previously for CD80 [30] A3Z1, MR and DC-SIGN expression was assessed with specific primers and SybrGreen Master Mix (Takara, Otsu, Japan) following conditions described previously [31].

β-actin expression was measured as a housekeeping gene, thus β-actin Ct values were subtracted from the markers’ Ct values for each sample, in order to obtain the 2^-ΔCt^ values used for comparisons.

### Viral infections and RT activity determination

Six-day stimulated MDM were infected at 0.1 TCID_50_/cell with one of the six SRLV strains under study, being one of genotype A (Ev1), three of genotype B (CAEV-Co, CAEV-To and 496) and two of the recently described genotype E of SRLV (Roccaverano and Seui). Following 2-h incubation, the cells were washed three times with PBS and fresh medium containing the stimulating cytokines was added. Supernatants were collected at 7 days post inoculation for Retrotranscriptase activity (RT activity) quantification, performed with the HS-Lenti RT activity kit (Cavidi, Uppsala, Sweden) according to the manufacturer’s instructions.

### Viral pseudotype constructions and entry assay

Plasmids containing the envelope gene (*env*) of Roccaverano, Seui [32] and Ev1 strains [33] were used. In addition, CAEV-Co *env* was cloned into the pCMV plasmid [34] using the same strategy and with the primer pairs shown in Table 1. Positive clones were screened by restriction enzyme digestion, sequencing and by assessing the formation of syncitya in cultured skin fibroblasts. pMDG plasmid encoding for vesicular stomatitis virus env glycoprotein (VSV-G) was used as a positive control (kindly provided by Prof. Greg J. Towers, University of London, UK).

For entry assays, pseudotyped virions were produced by co-transfection of HEK 293-T cells with two types of plasmids as described [32]. Briefly, pCAEV-AP (encoding alkaline phosphatase), kindly provided by Dr Isidro Hötzel, [35]; and one of the *env*-containing plasmid constructs described above. HEK 293-T cell culture supernatants containing the pseudotyped viruses were collected after 48 h, clarified and used in 10-fold dilutions to infect MDM, CHO and CHO-MR cells. After 48 h, the cells were stained using the alkaline phosphate substrate BCIP/NBT (Thermo Scientific) [36] and the results were expressed as focus-forming unit per mL (FFU/mL).

### Mannan treatment

Stimulated and pN3-stimulated MDM, CHO and CHO-MR cells were treated with 1 mg/mL of mannan (Sigma-Aldrich), for 30 min at 37 °C and 5% CO_2_, and infected with 10-fold dilutions of the pseudotyped viruses plus mannan following the entry assay procedure described above. Cells non-treated with mannan were included as controls.

### Virus-induced polarization

MDM were allowed to differentiate in complete medium without cytokine stimulation and then infected with 0.1 TCID_50_/mL of the six strains mentioned above (EV1, CAEV-Co, CAEV-To, 496, Roccaverano and Seui). On day 7 of infection, the cells were harvested and RNA were extracted and retrotranscribed to cDNA. Real time PCR for the detection of TNF-α, CD80, A3Z1, MR, DC-SIGN and IL-10, were carried out as described above.

### Macrophage versatility

MDM were obtained and allowed to differentiate into M1 or M2 profiles with IFN-γ and IL-4 respectively through two cycles of stimulation (3 days each). After the second, MDM were washed and stimulated for 3 additional days with a cytokine of the opposite phenotype (M1 vs. M2), then RNA was obtained and cDNA were subjected to specific amplification of differentiation pathway markers.

### Statistical analysis

The effects of different stimulators on marker expression were tested evaluating correlation between 2^-ΔCt^ and stimulator concentration with the Spearman’s rank correlation test. In order to evaluate the possible differences in expression among cells treated with different stimuli or non-treated (i.e. control pN3 group), data were analysed using Kruskal Wallis test. If a significant difference was recorded, a planned comparison between each treated cell group and the control was performed using 2-tailed Wilcoxon rank-sum test or Mann-Whitney’s test. Correlations and differences were considered significant if associated *p* < 0.05.

## Results

### Cytokine biological activity

**Figure 1:**
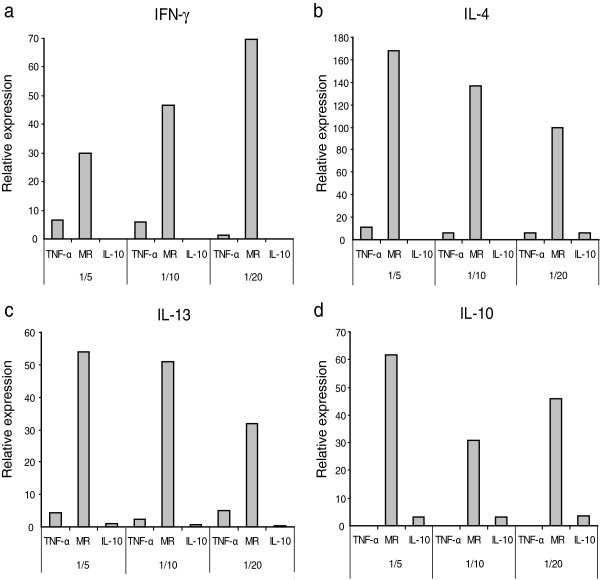
**Biological activity.** Supernatants containing caprine IFN-γ **(a)**, IL-4 **(b)**, IL-13 **(c)** and IL-10 **(d)** produced in HEK293-T cells, as described in Material and Methods were applied to MDM in serial dilutions (1/5, 1/10 and 1/20). Biological activity was evaluated as the relative expression of TNF-α, MR and IL-10 evaluated by real time RT-PCR. Values are expressed as 2^-ΔCt^ × 100 normalized to β-actin.

Supernatants of HEK-293-T cells transfected with IL-13 or IL-10 expression plasmids were found to affect MDM M1/M2 marker expression (quantified by real-time RT-PCR). Variation of M1/M2 markers was dose dependent (Figure 1). IL-13 favored MR expression since higher dilutions showed lower MR expression levels (Spearman Rho = 0.956, *p* < 0.005); similarly, TNF-α expression slightly increased with higher dilutions of IL-13 (Spearman Rho *p* < 0.005), but resulted in negligible variations. Even if the relationship is not linear, IL-10 showed as did IL-13 a positive relation with MR since the more cytokine is present the higher level of MR expression is observed.

Therefore these cytokines, in addition to those whose activity had been assessed by commercial kits (IFN-γ and IL-4), were considered biologically active and suitable for further MDM stimulation studies.

### Characterization and plasticity of polarized macrophages

Phenotypic differences regarding cell morphology (shape and size) were clearly observed between differentially stimulated MDM after 3 days of cytokine stimulation. Specifically, IFN-γ and LPS-treated (Th1 cytokines) cells were small and round, whereas IL-4 treated MDM showed a dendritic-like shape with large pseudopodia. Finally, in macrophages exposed to IL-13, IL-10 and pN3 there appeared to be a mixture of morphological phenotypes.

**Figure 2:**
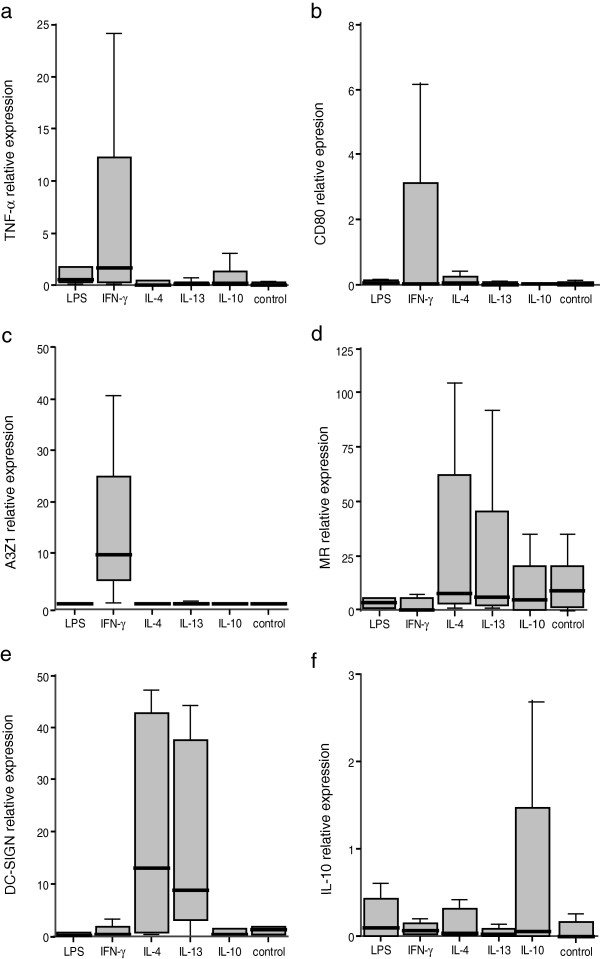
**Relative marker expression in stimulated MDM.** MDM were cultured in the presence of the particular cytokine or control (pN3). Relative expression of TNF-α **(a)**, CD80 **(b)**, A3Z1 **(c)**, MR **(d)**, DC-SIGN **(e)** and IL-10 **(f)** was measured by quantitative RT-PCR. The values are expressed as 2^-ΔCt^ × 100 median value (± interquartile range) of at least 3 independent experiments normalized to β-actin.

Phenotypic characterization was further evaluated after two rounds (3 days each) of exposure to LPS, IFN-γ, IL-4, IL-13 and IL-10, by assessing the expression of TNF-α, CD80 and A3Z1 (M1 phenotype) and MR, DC-SIGN and IL-10 (M2 phenotype) markers. Relative marker expression, measured by real time RT-PCR (Figure 2) indicated that, as described in other species, TNF-α expression increased in IFN-γ stimulated MDM from caprine and ovine origin and therefore this molecule could be a good marker for M1 polarization. In contrast, MR expression increased in IL-4 stimulated MDM and thus could be considered as a good marker for M2 polarization.

Additional molecules evaluated in this study were identified as new markers for M1 or M2 macrophage populations. A3Z1 and CD80 were induced in IFN-γ stimulated MDM, identifying M1 macrophages. Conversely, DC-SIGN was highly expressed in IL-4 and IL-13 stimulated macrophages, identifying M2 macrophages (Figure 2).

IL-10 expression values did not seem to distinguish between different populations (p > 0.05 in all comparisons), although expression was mainly observed in IL-4, IL-10 and LPS stimulated macrophages.

Apart from these clear patterns of M1 vs. M2 polarization, “single-effect” patterns associated to caprine or ovine species were also identified. Specifically, LPS stimulation resulted in highly increased levels of MR in goats and IL-10 in sheep and not in increased M1 cytokines, whereas IL-13 induced high levels of MR and DC-SIGN in both species. CD80 was a good marker for M1 macrophages in the caprine species whereas IL-4 stimulation led to expression of IL-10 only in sheep.

pN3 induced the lowest levels of stimulation in ovine and caprine macrophages. As in other animal models, pN3 (control) exposure resulted in increased levels of M2 marker expression.

**Figure 3:**
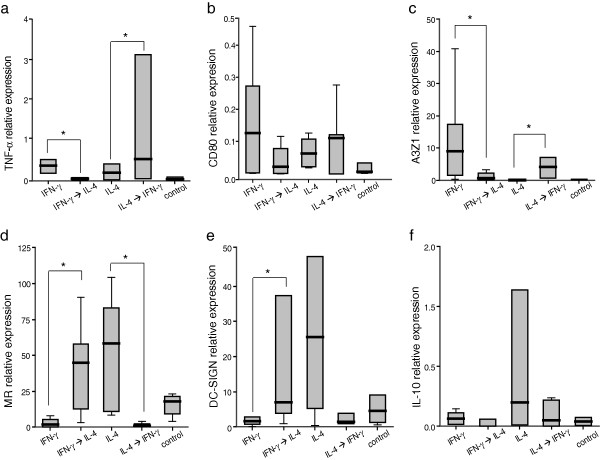
**Macrophage plasticity.** MDM were stimulated for a total of 9 days, 6 days with IFN-γ, IL-4 or pN3 (non-stimulated control) followed by a 3-day stimulation with the opposite cytokine as indicated with arrows. Relative expression of the markers TNF-α **(a)**, CD80 **(b)**, A3Z1 **(c)**, MR **(d)**, DC-SIGN **(e)**, and IL-10 **(f)** was quantified by real time RT-PCR after 6 days of total stimulation. The values are expressed as the median (± interquartile range) 2^-ΔCt^ × 100 value related to β-actin, and represent at least 3 independent experiments. *p < 0.05 (paired Wilcoxon Rank Sum test).

In order to explore whether M1 and M2 were irreversibly differentiated cells, ovine macrophages were polarized into M1 and M2 patterns by stimulation with IFN-γ and IL-4 respectively, and then stimulated with a cytokine of the opposite pattern (IL-4 and IFN-γ, respectively). When M1 (IFN-γ stimulated) macrophages expressing high levels of TNF-α and A3Z1 markers were stimulated with IL-4, expression of these markers was significantly reduced (paired Wilcoxon Rank Sum test *p* < 0.05 in both cases) while MR and DC-SIGN expression was increased (*p* < 0.05 in both cases, Figure 3). Similarly, when M2 (IL-4 stimulated) macrophages were stimulated with IFN-γ, their profile changed drastically towards phenotype M1 with increased expression of TNFα and A3Z1 (*p* < 0.05 in both cases) and significant reduction of MR expression (*p* < 0.05). DC-SIGN only showed a trend to lower expression (*p* = 0.1). These results indicate that the polarization profile of small ruminant MDM is plastic and M1 and M2 patterns defined here can be reverted from one to the other according to cytokine availability.

### Susceptibility of MDM stimulated with cytokines to virus infection

**Figure 4:**
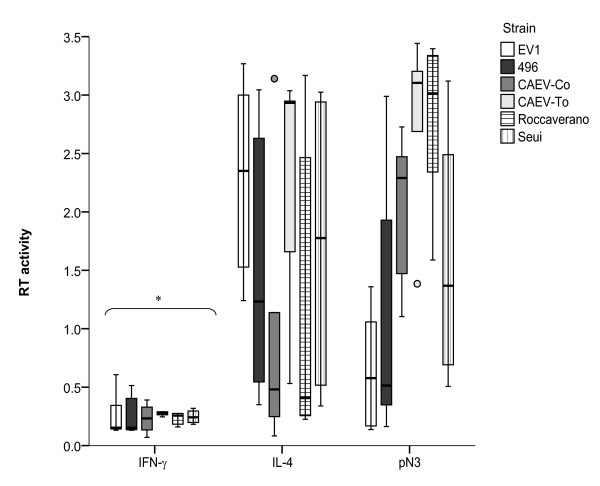
**SRLV replication in polarized macrophages.** MDM were exposed for 6 days to the cytokines IFN-γ, IL-4, or pN3 (non-stimulated control), and subsequently infected with different SRLV strains: EV1 (empty bars), 496 (full bars), CAEV-Cork (grey bars) CAEV-To (light grey bars), Roccaverano (horizontal-lined bars) and Seui (vertical-lined bars). RT activity was measured (A_450nm_) in clarified supernatants at 7 days post infection. The values are the median (± interquartile range) of at least 3 independent experiments. * *p* < 0.05 (paired Wilcoxon Rank Sum test).

Firstly, the MDM-SRLV combinations permissive to SRLV infection were chosen for infection after cytokine exposure. Combinations were designed including ovine macrophages and viral strains originally isolated from sheep, Ev1 (genotype A) or 496 (genotype B), and caprine macrophages and viral strains originally obtained from goats (CAEV-Co, CAEV-To, genotype B) or Roccaverano and Seui (genotype E). The corresponding MDM were submitted to two consecutive 3-day rounds of stimulation with cytokines, and then infected with different SRLV strains for 7 days to finally determine RT activity (Figure 4). The results indicate that M2 stimulated MDM when exposed to SRLV clearly favored viral replication. In contrast, M1 IFN-γ stimulated cells showed a reduced RT activity upon SRLV infection compared to cells exposed to pN3 or IL-4 (Wilcoxon Rank Sum test *p* < 0.05; Figure 4). pN3 stimulated cells showed high levels of viral replication compatible with an M2 phenotype as shown in Figure 3. Thus, the ability to support SRLV replication (pattern of RT activity) differed according to the stimulatory cytokine, with IFN-γ (M1 phenotype) efficiently inhibiting infection independently of the strain or the ruminant species from which MDM were isolated.

### SRLV infection promotes the M2 differentiation pathway

**Figure 5:**
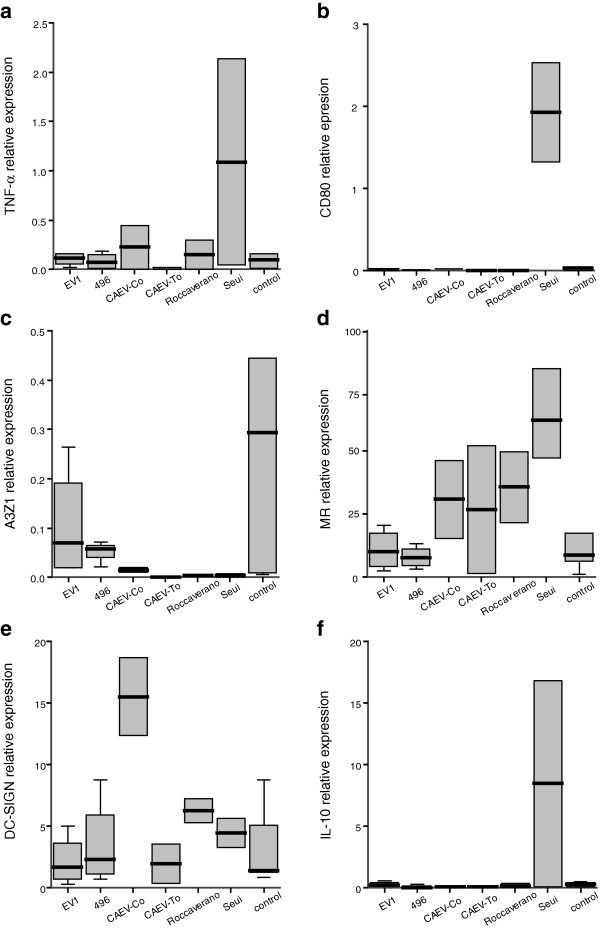
**Virus-induced polarization of MDM.** MDM were allowed to differentiate in the absence of cytokine and then infected with different SRLV strains (EV1, 496, CAEV-Cork, CAEV-To, Roccaverano and Seui) at a MOI of 0.1. Six day post-infection relative expression of the markers TNF-α **(a)**, CD80 **(b)**, A3Z1 **(c)**, MR **(d)**, DC-SIGN **(e)**, and IL-10 **(f)** was measured by quantitative RT-PCR. The values are expressed as 2^-ΔCt^ × 100, related to β-actin, and represent the median (± interquartile range) of at least 3 independent experiments.

Knowing that different cytokines trigger the establishment of different polarization patterns in small ruminant macrophages, we explored the hypothesis that SRLV would also induce a particular polarization pattern. For this and in the absence of cytokine stimulation, MDM were infected with different SRLV strains and differentiation markers quantified on day 7 of infection. All the SRLV strains used in this study induced upregulation of M2 phenotype markers, with increased MR and DC-SIGN expression values compared to those of pN3 treated (Figure 5). M2 pattern of differentiation was more evident when comparing M1 vs M2 markers since pN3 stimulation alone induced an M2 polarization (Figure 2). While M1 markers were induced to a maximum of 2-fold compared with the housekeeping gene, M2 marker induction was up to 75-times higher than that of β-actin (Figure 5).

In contrast, TNFα, CD80 and A3Z1 and IL-10 expression did not increase upon infection with most of the strains. Remarkably, infection with Seui strain induced higher TNFα, CD80 and IL-10 expression than pN3 treated cells (Figure 5).

### Env-mediated entry studies with pseudotyped viruses and MR involvement

**Figure 6:**
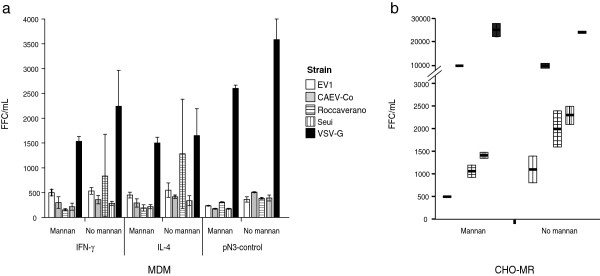
**Entry assay with pseudotyped viral particles.** IFN-γ, IL-4 and pN3 (control) stimulated MDM **(a)** and CHO-MR **(b)** were either treated or non-treated with mannan (1 mg/mL) before infection with pseudoviral particles bearing the envelope glycoproteins of the strains EV1 (empty boxes), CAEV-Cork (grey boxes), Roccaverano (horizontal-lined boxes), Seui (vertical-lined boxes) or the control VSV-G (full boxes). Foci of transduced cells were counted using alkaline phosphatase activity staining. Values are expressed as median focus-forming units per mL (FFC/mL) of at least 3 independent experiments.

Knowing, on the one hand, the capacity of SRLV to infect MDM and trigger the production of an M2 phenotype (above) and, on the other hand, the possible role of MR in SRLV entry and infection [31] and the increased production of MR upon IL-4 stimulation, we first studied env-mediated viral entry using for infection, pseudoviruses expressing the envelope protein of known SRLV strains (Ev1, CAEV-Co, Roccaverano, Seui) or VSV protein G (Figure 6a). The results indicate that viral entry into MDM was not affected by the particular macrophage phenotype (M1 or M2) since IFN-γ and IL-4 stimulated macrophages showed similar numbers of focus forming units with the different pseudotypes.

Viral entry was significantly inhibited by mannan treatment when using Roccaverano pseudotyped particles overall in IL-4 stimulated MDM, and the inhibitory effect was non-significant for Ev1, CAEV-Co, Seui or VSV-G pseudotyped virions. The highest entry values were reached with pantropic VSV-G pseudovirions, as expected (Figure 6a).

Next, we determined if the mannan blocking effects of env-mediated entry and subsequent infection could be attributed to the involvement of the MR receptor at viral entry, also using for this the pseudoviruses described above. For this we used CHO-MR cells stably expressing MR [24], but not other SRLV receptors, and assessed the effect of mannan treatment (Figure 6b). CHO-MR cells challenged with EV1, CAEV-Co, Roccaverano, Seui or VSV-G pseudotyped viruses showed a heterogeneous pattern of viral entry and suggest that mannan exposure would inhibit infections by Ev1, Roccaverano and Seui pseudotyped virions but did not hamper CAEV-Co or VSV-G entry (Figure 6b).

## Discussion

In SRLV infections, immune responses constitute a double-edge sword, since on one side they are essential against infections [37] and on the other side they lead to follicular hyperplasia and mononuclear cell infiltration, the main causes of tissue damage in SRLV-related lesions [11,38]. Macrophages, the main target cells for SRLV infection, play a pivotal role in the process, disseminating the virus and providing antigen, costimulatory signals and cytokines, inducers or regulators of immune responses. In addition, macrophages suffer alterations upon lentiviral infection, but subsets of macrophages have not been identified yet in small ruminant species. Our results, strongly suggest that M1 and M2, main patterns of differentiation, are well conserved among species - including small ruminants - being induced by IFN-γ and IL-4, respectively. In small ruminants, stimulation with M1-like stimulus (IFN-γ) clearly induced an increased expression of not only TNF-α, as in humans and mice [39] but also APOBEC3 (A3Z1) and the costimulatory molecule CD80, together with a decreased expression of MR and DC-SIGN. Conversely, stimulation with M2 stimulus (IL-4, IL-13, IL-10) conferred an induction of not only MR but also DC-SIGN and IL-10. Interestingly, LPS and negative controls (pN3) induced an intermediate (M1-to-M2) pattern and a clear M2 pattern, respectively. LPS has been associated with an M2b profile [19], with high levels of proinflammatory cytokines together with high IL-10 [40,41] as shown by our studies.

SRLV viral infection induced M2 polarization of macrophages, which also exhibited high MR and DC-SIGN expression as observed upon IL-4 stimulation. If this SRLV-mediated M2 induction occurs in vivo in alveolar macrophages of infected animals, infection would be enhanced, as these cells would show a M2 phenotype with a high expression of pattern-recognition receptors such as MR and scavenger receptors [42]. This pattern would favor viral spread as observed [43] and also in this study using M2 polarized macrophages. Ovine alveolar macrophages expressing scavenger receptor CD163, a hallmark of M2 differentiation, were found to be highly related to the presence of SRLV capsid protein in persistently infected sheep [44], in agreement with our results. However, during early infection in vivo, new M1 cells may infiltrate into the tissue and initially control the incoming virus. After encountering the virus (Figure 5) and also for physiological reasons (M1 cells last a few days; [45]), these M1 would eventually switch to M2 macrophages [46] allowing virus replication as well as infection spread since M2 cells can live from several weeks to years [47]. M1 cells are non-motile since inflammation is better tolerated in a restricted site, but M2 cells instead are motile (amoeboid and mesenchymal modes), able to move across a 3D matrix [48] favoring infection spread to other target organs. M2 cells are tolerogenic and surely are the main macrophages in SRLV infection since animals that reach the clinical stage present high levels of IgG1, IL-4 and low levels of B7 molecule transcripts that would impair antigenic presentation leading to anergic responses and T cell exhaustion. However, care should be taken when interpreting this picture since it is also plausible that M1 cells somehow impaired in their ability to restrict SRLV, could be responsible for both the tolerance and immunomediated lesions.

The Roccaverano SRLV strain showed an increased entry level into M2 macrophages compared to M1 suggesting a different receptor usage for genotype E1, whereby this genotype would enter the cell mainly via mannose recognizing receptors such as MR, whereas other strains would use additional receptors present in macrophages [31,32]. The levels of env-related entry into CHO-MR cells found in this study in the presence/absence of mannan may also suggest that SRLV can enter M2 cells via MR.

M2, the most susceptible macrophages for SRLV infection, could allow viral proliferation and promote pathogenesis in vivo by at least two different mechanisms. One pathway would involve cellular receptors that may allow or enhance viral entry, and the other related to poor expression of B7 (CD80/86) costimulatory molecules, also observed here in M2 profiles, which would lead to the lack of T cell responses in vivo [30]. Arguing also in favour of these mechanisms, are studies showing that IFN-γ (leading to the M1 phenotype) selectively down regulates activity of MR [49,50] and M1 chemokines amplify the DTH response, which is reduced in SRLV affected animals [51].

Interestingly, infection block (decreased RT-activity) in M1 compared to M2 MDM did not occur at the entry step, since the degree of entry was similar in M1 and M2 cells. Rather, blockage occurred at post-entry steps since preliminary qPCR data suggest that integration, evaluated as proviral load, was lower in IFN-γ than IL-4 stimulated cells (data not shown). Post-integration restriction may include the involvement of TRIM5 and APOBEC, as described in human HIV-1 infections [22]. M1 and M2 human macrophages showed restriction against HIV-1 infection by inducing factors like APOBEC3G or reducing the expression of certain receptors (CD4) respectively [22]. In this study, M1 differentiation may have restricted SRLV replication through A3Z1 induction, according to the A3Z1 mRNA levels observed. Conversely, M2 favoured virus replication likely due to differential receptor usage of SRLV compared with HIV-1.

Macrophages respond to stimuli temporarily reverting their differentiation pattern, depending on the cytokine present [52,53]. Thus polarization is a continuous reversible M1/M2 differentiation process, as confirmed in ovine macrophages, reflecting that profiles are not static terminally-differentiated states. This plasticity may have important consequences in chronic lentiviral infections where SRLV replicates at higher levels in M2 compared to M1 macrophages. Different strategies can be proposed in order to block or at least relieve M2 differentiation and therefore viral spread, since macrophages do not necessarily suffer apoptosis or emigration after inflammation [54,55]. These strategies have been applied against tumour diseases, where an M2 phenotype of macrophages may predominate [56] and the cure has involved a switch of the immune response to M1 by pharmacological treatment [57].

Interestingly, macrophages previously exposed to rhinoviruses show a reduced antibacterial capacity [58] and also SRLV infected macrophages showed a reduced phagocytic activity against bacteria indirectly facilitating secondary infections [59] suggesting the induction of an M2 phenotype. Mycoplasma co-infection malp (macrophage activation lipoprotein) may modify proteins presented on the plasmatic membrane that could act as SRLV (co)-receptors and favour infection [60]. Essentially, data regarding macrophage differentiation and characterization of the respective profiles share several features with the well depicted picture in humans and mice strongly suggesting a widely conserved maturation pathway, further reinforcing the M1/M2 model and validating their use.

## Competing interests

The authors declare that they have no competing interests.

## Authors’ contributions

HC and LB conducted the experiments and participated in the drafting of the paper and figures. LB also carried out statistical analysis. MJ was involved in macrophage isolation and stimulation, entry assays and RT activity determinations. IG was involved in macrophage isolation and APOBEC3Z1 relative expression determinations. BA and DA participated in the experimental design and search for funding resources. BA was also involved in the writing of the manuscript. SR and RR conceived and designed this study also being involved in the writing of the manuscript. All authors read and approved the final manuscript.

## References

[B1] Olech M Rachid A Croise B Kuzmak J Valas S Genetic and antigenic characterization of small ruminant lentiviruses circulating in Poland Virus Res 2011 163 528 536 22155513 10.1016/j.virusres.2011.11.019

[B2] L’Homme Y Ouardani M Levesque V Bertoni G Simard C Pisoni G Molecular characterization and phylogenetic analysis of small ruminant lentiviruses isolated from Canadian sheep and goats Virol J 2011 8 271 10.1186/1743-422X-8-271 21639904 PMC3123287

[B3] Kuhar U Barlic-Maganja D Grom J Phylogenetic analysis of small ruminant lentiviruses detected in Slovenia Vet Microbiol 2013 162 201 206 10.1016/j.vetmic.2012.08.024 23022680

[B4] Baryshnikova EI Malogolovkin AS Kolbasova OL Tsybanov S Comparative characteristics of the biological properties of small ruminant lentiviruses Vopr Virusol 2011 56 42 45 (in Russian) 21899070

[B5] Grego E Bertolotti L Quasso A Profiti M Lacerenza D Muz D Rosati S Genetic characterization of small ruminant lentivirus in Italian mixed flocks: evidence for a novel genotype circulating in a local goat population J Gen Virol 2007 88 3423 3427 10.1099/vir.0.83292-0 18024912

[B6] Reina R Bertolotti L Dei Giudici S Puggioni G Ponti N Profiti M Patta C Rosati S Small ruminant lentivirus genotype E is widespread in Sarda goat Vet Microbiol 2010 144 24 31 10.1016/j.vetmic.2009.12.020 20060658

[B7] Bertolotti L Mazzei M Puggioni G Carrozza ML Dei Giudici S Muz D Juganaru M Patta C Tolari F Rosati S Characterization of new small ruminant lentivirus subtype B3 suggests animal trade within the Mediterranean Basin J Gen Virol 2011 92 1923 1929 10.1099/vir.0.032334-0 21562119

[B8] Narayan O Kennedy-Stoskopf S Sheffer D Griffin DE Clements JE Activation of caprine arthritis-encephalitis virus expression during maturation of monocytes to macrophages Infect Immun 1983 41 67 73 6862634 10.1128/iai.41.1.67-73.1983 PMC264744

[B9] Ryan S Tiley L McConnell I Blacklaws B Infection of dendritic cells by the Maedi-Visna lentivirus J Virol 2000 74 10096 10103 10.1128/JVI.74.21.10096-10103.2000 11024138 PMC102048

[B10] Eriksson K McInnes E Ryan S Tonks P McConnell I Blacklaws B CD4(+) T-cells are required for the establishment of maedi-visna virus infection in macrophages but not dendritic cells in vivo Virology 1999 258 355 364 10.1006/viro.1999.9711 10366572

[B11] Blacklaws BA Small ruminant lentiviruses: immunopathogenesis of visna-maedi and caprine arthritis and encephalitis virus Comp Immunol Microbiol Infect Dis 2012 35 259 269 10.1016/j.cimid.2011.12.003 22237012

[B12] Kennedy PG Narayan O Ghotbi Z Hopkins J Gendelman HE Clements JE Persistent expression of Ia antigen and viral genome in visna-maedi virus-induced inflammatory cells. Possible role of lentivirus-induced interferon J Exp Med 1985 162 1970 1982 10.1084/jem.162.6.1970 2415661 PMC2187991

[B13] Juste RA Ott TL Kwang J Bazer FW de La Concha-Bermejillo A Effects of recombinant ovine interferon-tau on ovine lentivirus replication and progression of disease J Gen Virol 2000 81 525 532 10644852 10.1099/0022-1317-81-2-525

[B14] Zink MC Narayan O Kennedy PG Clements JE Pathogenesis of visna/maedi and caprine arthritis-encephalitis: new leads on the mechanism of restricted virus replication and persistent inflammation Vet Immunol Immunopathol 1987 15 167 180 10.1016/0165-2427(87)90110-3 3039719

[B15] Legastelois I Cordier G Cozon G Cadore JL Guiguen F Greenland T Mornex JF Visna-maedi virus-induced expression of interleukin-8 gene in sheep alveolar cells following experimental in vitro and in vivo infection Res Virol 1996 147 191 197 10.1016/0923-2516(96)80234-2 8901439

[B16] Zhang Z Harkiss GD Hopkins J Woodall CJ Granulocyte macrophage colony stimulating factor is elevated in alveolar macrophages from sheep naturally infected with maedi-visna virus and stimulates maedi-visna virus replication in macrophages in vitro Clin Exp Immunol 2002 129 240 246 10.1046/j.1365-2249.2002.01826.x 12165079 PMC1906446

[B17] Sharmila C Williams JW Reddy PG Effect of caprine arthritis-encephalitis virus infection on expression of interleukin-16 in goats Am J Vet Res 2002 63 1418 1422 10.2460/ajvr.2002.63.1418 12371770

[B18] Woodall CJ Maclaren LJ Watt NJ Differential levels of mRNAs for cytokines, the interleukin-2 receptor and class II DR/DQ genes in ovine interstitial pneumonia induced by maedi visna virus infection Vet Pathol 1997 34 204 211 10.1177/030098589703400305 9163876

[B19] Mantovani A Sica A Sozzani S Allavena P Vecchi A Locati M The chemokine system in diverse forms of macrophage activation and polarization Trends Immunol 2004 25 677 686 10.1016/j.it.2004.09.015 15530839

[B20] Vacca P Cantoni C Vitale M Prato C Canegallo F Fenoglio D Ragni N Moretta L Mingari MC Crosstalk between decidual NK and CD14+ myelomonocytic cells results in induction of Tregs and immunosuppression Proc Natl Acad Sci U S A 2010 107 11918 11923 10.1073/pnas.1001749107 20547831 PMC2900704

[B21] Gordon S The macrophage: past, present and future Eur J Immunol 2007 37 Suppl 1 S9 S17 17972350 10.1002/eji.200737638

[B22] Cassol E Cassetta L Rizzi C Alfano M Poli G M1 and M2a polarization of human monocyte-derived macrophages inhibits HIV-1 replication by distinct mechanisms J Immunol 2009 182 6237 6246 10.4049/jimmunol.0803447 19414777

[B23] Mantovani A Biswas SK Galdiero MR Sica A Locati M Macrophage plasticity and polarization in tissue repair and remodelling J Pathol 2013 229 176 185 10.1002/path.4133 23096265

[B24] Martinez-Pomares L Reid DM Brown GD Taylor PR Stillion RJ Linehan SA Zamze S Gordon S Wong SY Analysis of mannose receptor regulation by IL-4, IL-10, and proteolytic processing using novel monoclonal antibodies J Leukoc Biol 2003 73 604 613 10.1189/jlb.0902450 12714575

[B25] Sargan DR Bennet ID Cousens C Roy DJ Blacklaws BA Dalziel RG Watt NJ McConnell I Nucleotide sequence of EV1, a British isolate of maedi-visna virus J Gen Virol 1991 72 1893 1903 10.1099/0022-1317-72-8-1893 1651983

[B26] Saltarelli M Querat G Konings DA Vigne R Clements JE Nucleotide sequence and transcriptional analysis of molecular clones of CAEV which generate infectious virus Virology 1990 179 347 364 10.1016/0042-6822(90)90303-9 2171210

[B27] Grego E Profiti M Giammarioli M Giannino L Rutili D Woodall C Rosati S Genetic heterogeneity of small ruminant lentiviruses involves immunodominant epitope of capsid antigen and affects sensitivity of single-strain-based immunoassay Clin Diagn Lab Immunol 2002 9 828 832 12093681 10.1128/CDLI.9.4.828-832.2002 PMC120019

[B28] Glaria I Reina R Crespo H de Andrés X Ramirez H Biescas E Pérez MM Badiola J Lujan L Amorena B de Andrés D Phylogenetic analysis of SRLV sequences from an arthritic sheep outbreak demonstrates the introduction of CAEV-like viruses among Spanish sheep Vet Microbiol 2009 138 156 162 10.1016/j.vetmic.2009.03.002 19339126

[B29] Reina R Barbezange C Niesalla H de Andrés X Arnarson H Biescas E Mazzei M Fraisier C McNeilly TN Liu C Perez M Carrozza ML Bandecchi P Solano C Crespo H Glaria I Huard C Shaw DJ de Blas I de Andrés D Tolari F Rosati S Suzan-Monti M Andrésdottir V Torsteinsdottir S Petursson G Lujan L Pepin M Amorena B Blacklaws B Mucosal immunization against ovine lentivirus using PEI-DNA complexes and modified vaccinia Ankara encoding the gag and/or env genes Vaccine 2008 26 4494 4505 10.1016/j.vaccine.2008.06.065 18606204

[B30] Reina R Glaria I Benavides J de Andrés X Crespo H Solano C Pérez V Lujan L Pérez MM de la Lastra JM P Rosati S Blacklaws B Harkiss G de Andrés D Amorena B Association of CD80 and CD86 expression levels with disease status of Visna/Maedi virus infected sheep Viral Immunol 2007 20 609 622 10.1089/vim.2007.0071 18158734

[B31] Crespo H Reina R Glaria I Ramirez H de Andrés X Jauregui P Lujan L Martinez-Pomares L Amorena B de Andrés DF Identification of the ovine mannose receptor and its possible role in Visna/Maedi virus infection Vet Res 2011 42 28 10.1186/1297-9716-42-28 21314911 PMC3041668

[B32] Juganaru M Reina R Bertolotti L Stella MC Profiti M Armentano M Bollo E Amorena B Rosati S In vitro properties of small ruminant lentivirus genotype E Virology 2011 410 88 95 10.1016/j.virol.2010.10.031 21094509

[B33] Sanchez AB Rodriguez D Garzon A Amorena B Esteban M Rodriguez JR Visna/maedi virus Env protein expressed by a vaccinia virus recombinant induces cell-to-cell fusion in cells of different origins in the apparent absence of Env cleavage: role of glycosylation and of proteoglycans Arch Virol 2002 147 2377 2392 10.1007/s00705-002-0874-7 12491104

[B34] Hotzel I Cheevers WP Host range of small-ruminant lentivirus cytopathic variants determined with a selectable caprine arthritis- encephalitis virus pseudotype system J Virol 2001 75 7384 7391 10.1128/JVI.75.16.7384-7391.2001 11462010 PMC114973

[B35] Hotzel I Cheevers W Differential receptor usage of small ruminant lentiviruses in ovine and caprine cells: host range but not cytopathic phenotype is determined by receptor usage Virology 2002 301 21 31 10.1006/viro.2002.1575 12359443

[B36] Miller’s Lab http://www.protocol-online.org/cgi-bin/prot/view_cache.cgi?ID=3479

[B37] Appay V Sauce D Immune activation and inflammation in HIV-1 infection: causes and consequences J Pathol 2008 214 231 241 10.1002/path.2276 18161758

[B38] Bertoni G Blacklaws B Desport ME Small ruminant lentiviruses and cross species transmission Lentiviruses and Macrophages: Molecular and Cellular interactions 2010 Norfolk, UK: Caister Academic Press 277 306

[B39] Martinez FO Gordon S Locati M Mantovani A Transcriptional profiling of the human monocyte-to-macrophage differentiation and polarization: new molecules and patterns of gene expression J Immunol 2006 177 7303 7311 17082649 10.4049/jimmunol.177.10.7303

[B40] Mosser DM The many faces of macrophage activation J Leukoc Biol 2003 73 209 212 10.1189/jlb.0602325 12554797

[B41] Sica A Mantovani A Macrophage plasticity and polarization: in vivo veritas J Clin Invest 2012 122 787 795 10.1172/JCI59643 22378047 PMC3287223

[B42] Gordon S Martinez FO Alternative activation of macrophages: mechanism and functions Immunity 2010 32 593 604 10.1016/j.immuni.2010.05.007 20510870

[B43] Crespo H Jauregui P Glaria I Sanjose L Polledo L Garcia-Marin JF Lujan L de Andrés D Amorena B Reina R Mannose receptor may be involved in small ruminant lentivirus pathogenesis Vet Res 2012 43 43 10.1186/1297-9716-43-43 22591485 PMC3497593

[B44] Herrmann-Hoesing LM Noh SM Snekvik KR White SN Schneider DA Truscott T Knowles DP Ovine progressive pneumonia virus capsid antigen as found in CD163- and CD172a-positive alveolar macrophages of persistently infected sheep Vet Pathol 2010 47 518 528 10.1177/0300985809359605 20382821

[B45] Bellingan GJ Caldwell H Howie SE Dransfield I Haslett C In vivo fate of the inflammatory macrophage during the resolution of inflammation: inflammatory macrophages do not die locally, but emigrate to the draining lymph nodes J Immunol 1996 157 2577 2585 8805660

[B46] Obot CJ Morandi MT Beebe TP Hamilton RF Holian A Surface components of airborne particulate matter induce macrophage apoptosis through scavenger receptors Toxicol Appl Pharmacol 2002 184 98 106 10.1006/taap.2002.9493 12408954

[B47] Murphy J Summer R Wilson AA Kotton DN Fine A The prolonged life-span of alveolar macrophages Am J Respir Cell Mol Biol 2008 38 380 385 10.1165/rcmb.2007-0224RC 18192503 PMC2274942

[B48] Bozinovski S Jones JE Vlahos R Hamilton JA Anderson GP Granulocyte/macrophage-colony-stimulating factor (GM-CSF) regulates lung innate immunity to lipopolysaccharide through Akt/Erk activation of NFkappa B and AP-1 in vivo J Biol Chem 2002 277 42808 42814 10.1074/jbc.M207840200 12208854

[B49] Whyte CS Bishop ET Ruckerl D Gaspar-Pereira S Barker RN Allen JE Rees AJ Wilson HM Suppressor of cytokine signaling (SOCS)1 is a key determinant of differential macrophage activation and function J Leukoc Biol 2011 90 845 854 10.1189/jlb.1110644 21628332

[B50] Liu Y Stewart KN Bishop E Marek CJ Kluth DC Rees AJ Wilson HM Unique expression of suppressor of cytokine signaling 3 is essential for classical macrophage activation in rodents in vitro and in vivo J Immunol 2008 180 6270 6278 18424750 10.4049/jimmunol.180.9.6270

[B51] Pyrah IT Watt NJ Immunohistological study of the depressed cutaneous DTH response in sheep naturally infected with an ovine lentivirus (Maedi-Visna virus) Clin Exp Immunol 1996 104 32 36 10.1046/j.1365-2249.1996.d01-661.x 8603529 PMC2200382

[B52] Duluc D Corvaisier M Blanchard S Catala L Descamps P Gamelin E Ponsoda S Delneste Y Hebbar M Jeannin P Interferon-gamma reverses the immunosuppressive and protumoral properties and prevents the generation of human tumor-associated macrophages Int J Cancer 2009 125 367 373 10.1002/ijc.24401 19378341

[B53] Stout RD Jiang C Matta B Tietzel I Watkins SK Suttles J Macrophages sequentially change their functional phenotype in response to changes in microenvironmental influences J Immunol 2005 175 342 349 15972667 10.4049/jimmunol.175.1.342

[B54] Stout RD Suttles J Immunosenescence and macrophage functional plasticity: dysregulation of macrophage function by age-associated microenvironmental changes Immunol Rev 2005 205 60 71 10.1111/j.0105-2896.2005.00260.x 15882345 PMC1201508

[B55] Issazadeh S Navikas V Schaub M Sayegh M Khoury S Kinetics of expression of costimulatory molecules and their ligands in murine relapsing experimental autoimmune encephalomyelitis in vivo J Immunol 1998 161 1104 1112 9686568

[B56] Niino D Komohara Y Murayama T Aoki R Kimura Y Hashikawa K Kiyasu J Takeuchi M Suefuji N Sugita Y Takeya M Ohshima K Ratio of M2 macrophage expression is closely associated with poor prognosis for Angioimmunoblastic T-cell lymphoma (AITL) Pathol Int 2010 60 278 283 10.1111/j.1440-1827.2010.02514.x 20403029

[B57] Guiducci C Vicari AP Sangaletti S Trinchieri G Colombo MP Redirecting in vivo elicited tumor infiltrating macrophages and dendritic cells towards tumor rejection Cancer Res 2005 65 3437 3446 15833879 10.1158/0008-5472.CAN-04-4262

[B58] Oliver BG Lim S Wark P Laza-Stanca V King N Black JL Burgess JK Roth M Johnston SL Rhinovirus exposure impairs immune responses to bacterial products in human alveolar macrophages Thorax 2008 63 519 525 10.1136/thx.2007.081752 18245149

[B59] Monleon E Pacheco MC Lujan L Bolea R Luco DF Vargas MA Alabart JL Badiola JJ Amorena B Effect of in vitro maedi-visna virus infection on adherence and phagocytosis of staphylococci by ovine cells Vet Microbiol 1997 57 13 28 10.1016/S0378-1135(97)00080-1 9231978

[B60] Rosati S Pozzi S Robino P Montinaro B Conti A Fadda M Pittau M P48 major surface antigen of Mycoplasma agalactiae is homologous to a malp product of Mycoplasma fermentans and belongs to a selected family of bacterial lipoproteins Infect Immun 1999 67 6213 6216 10531294 10.1128/iai.67.11.6213-6216.1999 PMC97020

